# Genotype-Phenotype of CRB1-Associated Early-Onset Retinal Dystrophy: Novel Insights on Retinal Architecture and Therapeutic Window for Clinical Trials

**DOI:** 10.1167/iovs.65.3.11

**Published:** 2024-03-11

**Authors:** Yili Jin, Songshan Li, Zhaoxin Jiang, Limei Sun, Li Huang, Ting Zhang, Xinyu Liu, Xiaoyan Ding

**Affiliations:** 1 State Key Laboratory of Ophthalmology, Zhongshan Ophthalmic Center, Sun Yat-Sen University, Guangdong Provincial Key Laboratory of Ophthalmology and Visual Science, Guangzhou, China; 2 Guangdong Provincial Clinical Research Center for Ocular Diseases, Guangzhou, China

**Keywords:** crumbs homolog 1 (*CRB1*), early onset severe retinal dystrophy (EOSRD), swept-source optical coherence tomography (SS-OCT), inner/outer (I/O) ratio

## Abstract

**Purpose:**

The purpose of this study was to investigate the genotypic and phenotypic characteristics of CRB1-associated early onset retinal dystrophy (CRB1-eoRD) and retinal architecture by swept-source optical coherence tomography (SS-OCT).

**Methods:**

Eleven probands with CRB1-eoRD were recruited. Clinical information, genetic analysis, and comprehensive ophthalmic examinations including SS-OCT and SS-OCT angiography (SS-OCTA) were conducted.

**Results:**

A total of 81.8% (9/11) of CRB1-eoRD presented as Leber congenital amaurosis (LCA). Common clinical manifestations included coin-like yellow-white retinal spots (20/22, 90.9%) and para-arteriolar retinal pigment epithelial retention (12/22, 54.5%). Nineteen different *CRB1* variants were detected in our case series, including 12 missense, 3 frameshifts, 3 nonsense, and 1 splicing. Of them, 12 variants had been reported, and 7 were novel. SS-OCT showed thinner central macula (the LCA group, *P* < 0.0001), thicker total retina (*P* < 0.0001), thinner outer retina (*P* < 0.05), and thicker inner retina (*P* < 0.0001) compared with the healthy control. The inner/outer (I/O) retina thickness ratio of CRB1-eoRD was 3.0, higher than the healthy control of 1.2 and other inherited retinal diseases (IRDs) of 2.2 (*P* < 0.0001 and *P =* 0.0027, respectively). SS-OCTA revealed an increased vascular density and perfusion area of the superficial vascular complex and deep vascular complex in CRB1-eoRD.

**Conclusions:**

LCA emerges as a frequently occurring phenotype in CRB1-eoRD. The unique features of SS-OCT and SS-OCTA are illustrated, and the novel biomarker, I/O ratio, may facilitate early diagnosis. The insights gained from this study hold significant value in determining the treatment window for potential forthcoming *CRB1* gene therapy.

Crumbs homolog 1 gene (*CRB1*; OMIM# 604210) mutation, inherited in an autosomal recessive pattern, is one of the common causes of retinal dystrophy, included type 8 Leber congenital amaurosis (LCA8; OMIM# 613835), early onset severe retinal dystrophy (EOSRD), type 12 retinitis pigmentosa (RP12; OMIM# 600105) and macular dystrophy (MD).^1^ *CRB1* mutations account for 7% to 17% LCA and 3% autosomal recessive RP.^2^^–^^5^

CRB1 is one of the human homologs of the Drosophila Crumbs. In humans, the *CRB1* gene is located on the long arm of chromosome 1 (1q31.1).^6^ It encodes a transmembrane protein with 1406 amino acids, which is located in the subapical region of Muller cells and photoreceptors and is one of the important components of the core CRB complex.^7^ The mutation of the *CRB1* gene will lead to abnormal function of the core CRB complex, which disrupts the normal polarity and adhesion functions of retinal cells. This disruption, in turn, results in abnormal retinal architecture, such as the development of retinoschisis, thinning of the outer retinal layer, and thickening of the inner retinal layer.^8^^–^^10^

Recently, *CRB1*-related gene therapy via subretinal injection has been proven to be effective in rat models^11^ and launched clinical trials.^12^ It is significant to understand the natural history, clinical heterogeneity, and the correlation between genes and phenotypes of CRB1-RD for selecting appropriate patients, treatment time points, and end points. At present, some studies have reported the clinical characteristics and natural history of CRB1-RD with a mean age between 16.2 and 38.7 years old.^2^^–^^5^^,^^13^ However, there have been no reports that specifically concentrate on elucidating the clinical characteristics of early-onset CRB1-related retinal dystrophy (CRB1-eoRD).

Herein, we present the study of 11 Han Chinese children with early-onset CRB1-RD (CRB1-eoRD) from 11 unrelated families, and we identified novel swept-source optical coherence tomography (SS-OCT) and SS-OCT angiography (SS-OCTA) features and discovered novel pathogenic *CRB1* mutations. The aim of this study is to report the genetic and clinical features, especially the retinal microstructure via SS-OCT of CRB1-eoRD.

## Materials and Methods

### Patient Selection and Genetics

We searched the pediatric inherited retinal dystrophy database in the Zhongshan Ophthalmologic Center and included patients with homozygous and compound heterozygous *CRB1* variants in children ≤9 years. This study was conducted following the tenets of the Declaration of Helsinki and was approved by the Medical Ethics Committee of the Zhongshan Ophthalmic Center, Sun Yat-sen University (2020KYPJ173-2). Informed written consent was obtained from the legal guardians of the patients.

The genomic DNA of the proband and available immediate family members was extracted from peripheral blood using the TIANamp Blood DNA Kit (DP348-03; Tiangen Biotech, Beijing, China), as instructed by the manufacturer. The quantity and quality of DNA were verified by using NanoDrop. Whole-exome sequencing (WES) was performed for 11 probands, and identified mutations were validated using Sanger sequencing of the family members. The Human Gene Mutation Database, Genome Aggregation Database, and Exome Aggregation Consortium were used to identify the reported pathogenic variants. Online algorithms, including MutationTaster, sorting intolerant from tolerant (SIFT), Polymorphism Phenotyping version 2 (Polyphen2), Protein Variation Effect Analyzer (PROVEN), and Combined Annotation-Dependent Depletion (CADD) and American College of Medical Genetics standard^14^ were used to evaluate the pathogenicity of mutations.

Clinical information, including age, gender, medical history, family history, symptoms, and clinical diagnosis were recorded. Standardized retrospective analysis of existing medical records was performed for data collection of the best corrected visual acuity (BCVA; LogMAR), refractive status (diopter [D]), slit lamp biomicroscopy, wide field scanning laser ophthalmoscope (Optomap 200Tx; Optos plc, Dunfermline, UK); As the traditional full-field ERGs (fERG) is difficult for young children to cooperate, the RETeval system (LKC Technologies, Gaithersburg, MD, USA) was used in all children for fERG examination. This system used skin electrodes and was conducted without pupil dilation.^15^ The International Society for Clinical Electrophysiology of Vision (ISCEV) standards were followed for the fERG measurement.^16^

### SS-OCT and SS-OCTA Analysis

**Figure 1.**
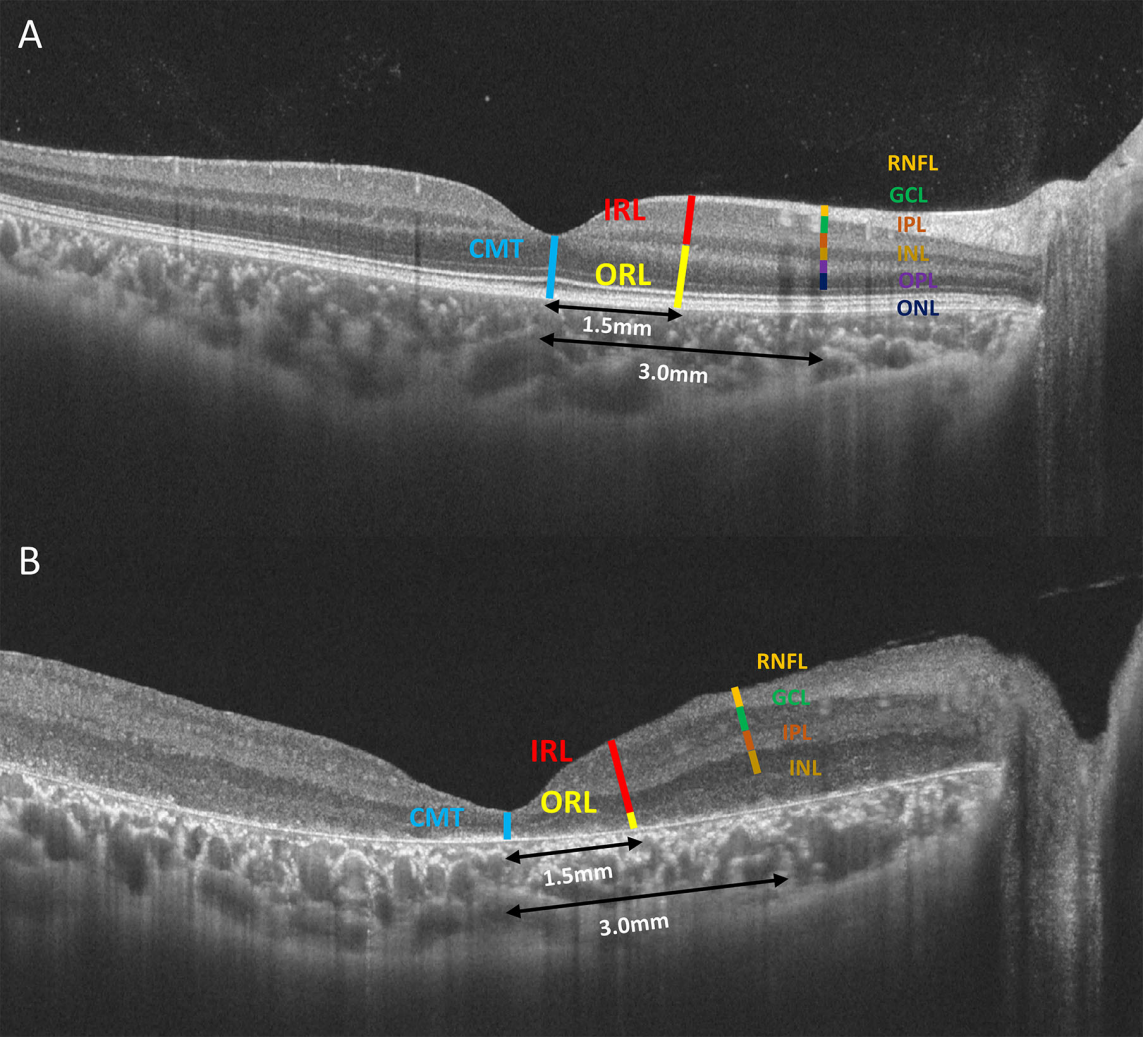
Diagram of measurement pattern in SS-OCT. The healthy control (**A**) and CRB1-eoRD (**B**) were manually measured at central macula, 1.5 mm and 3.0 mm to the central macula.

VG200S (SVision Imaging, Henan, China) is a new SS-OCT and SS-OCTA technology that uses a 1050 nm length light source, tracking system, and can conduct 2 × 10^5^ A-scans per second. VG200S was used to perform and analyze the retinal microstructure as previously reported.^17^^–^^19^ In our study, the macular structure was assessed using the Macular Raster mode on the SS-OCT machine, which involved the acquisition of 33 Horizontal B scans. To ensure precision and consistency, we carefully selected the B-scan containing the macular foveola for manual measurements. Each image was measured three times, and the resulting measurements were averaged. Additionally, we incorporated a built-in tracking system to mitigate any potential effects of eye movement during the imaging and measurement process. The integrity of outer nuclear layer (ONL), external limiting membrane (ELM), ellipsoid zone (EZ), and retinal pigment epithelium (RPE) were evaluated. The ONL was defined as totally atrophic, residual macula, thinning, and fovea cystoid changes. The ELM, EZ, and RPE were defined as intact, interrupted, or unrecognizable. We manually measured (Fig. 1) central macular thickness (CMT), outer retinal thickness (ORT; upper bound of outer plexiform layer to RPE), inner retinal thickness (IRT; internal limiting membrane to lower bound of inner nuclear layer), retinal nerve fiber layer (RNFL), ganglion cell layer (GCL), inner plexiform layer (IPL), and inner nuclear layer (INL) on the fovea, nasal retina (1.5 mm nasal to macular, labeled as N_1.5_; and 3.0 mm nasal to macular, labeled as N_3.0_) and temporal retina (1.5 mm temporal to macular, labeled as T_1.5_; and 3.0 mm temporal to macular, labeled as T_3.0_). We included 20 age and gender-matched healthy children and 11 age-matched inherited retinal diseases (IRDs; including individuals with Stargardt's disease carrying ABCA4 variants, as well as individuals with Cone-Rod Dystrophy or RP presenting with various genetic variants such as RDH12, RPGR, LCA5, CEP290, RP2, USH2A, and EYS) but without CRB1 variant as the control group. SS-OCT images were assessed by two authors and reviewed by a third author in case of discrepancy between the two authors. The 6 × 6 mm SS-OCTA scans centered on the fovea were obtained using the tracking system for both eyes. Build-in software was used to analyze the vascular density and perfusion area of the superficial vascular complex (SVC) and deep vascular complex (DVC).

### Statistics

Data analysis was carried out with Excel 2019 (Microsoft, Redmond, WA, USA) and GraphPad Prism 8.0.2 (GraphPad Software, San Diego, CA, USA). All quantitative data were expressed as mean ± standard deviation. The normality of data was analyzed using the Shapiro-Wilk test. Comparisons of three groups continuous variables were performed using 1-way analysis of variance (ANOVA) and Bonferroni multiple comparison. The threshold of significance for all statistical tests was set at *P* < 0.05.

## Results

### Clinical Features

Eleven children (22 eyes) from 11 unrelated Chinese Han families were recruited. The gender ratio was 7 and 4 (boys and girls), and the mean age was only 4.82 ± 1.62 years old. Based on the clinical manifestation, nine children were diagnosed as LCA, and two children as non-LCA. The two non-LCA children presented with macular schisis with paravascular changes, who were both diagnosed as intermediate uveitis at the first visit and were eventually diagnosed as RP. The mean BCVA was 1.50 ± 0.87 LogMAR in 9 children, whereas it was unrecordable in 2 children, one a 1-year 8-month-old girl and another 3-year 6-month boy both with nystagmus. The mean equivalent spherical was +6.41 ± 3.76 D. The fmean onset age of CRB1-eoRD is 2.85 ± 2.45 years (2.17 ± 2.15 years in the LCA group and 5.92 ± 0.91 years in the non-LCA group). The mean disease duration of eo-CRB1 is 1.82 ± 1.86 years, the LCA group is 2.10 ± 1.94 years, the non-LCA is 0.58 ± 0.42 years. The detailed clinical manifestations of 11 probands were shown in Table 1.

**Table 1.** Clinical Characteristics of CRB1-Associated Early Onset Retinal Dystrophy


**Characteristics**	
Patients	11
Eyes	22
Gender ratio (M/F)	7/4
Age, y	4.82 ± 1.62 (1.67–7.0)
BCVA (LogMAR)^*^	1.50 ± 0.87 (0.16–2.8)
Spherical equivalent (D) for CRB1	+6.41 ± 3.76 (−0.75 to +12.5)
Spherical equivalent (D) for control	+0.36 ± 1.92 (−7.00 to +5.25)
**Classification**	
LCA	9/11 (81.8%)
Non-LCA^#^	2/11 (18.2%)
**Age of onset of disease** **,** **y**	
Total	2.85 ± 2.45
LCA	2.17 ± 2.15
Non-LCA^#^	5.92 ± 0.91
**Disease duration** **,** **y**	
Total	1.82 ± 1.86
LCA	2.10 ± 1.94
Non-LCA^#^	0.58 ± 0.42
**Signs** **(eyes)**	
PPRPE	12/22 (54.5%)
Coin-like yellow-white retinal spots	20/22 (90.9%)
Macular edema	6/22 (27.3%)
Macular atrophy	13/22 (59.1%)
Coats-like	1/22 (4.5%)
FFA leakage^✝^	8/8 (100%)

F, female; FFA, fluorescein fundus angiography; LCA, Leber congenital amaurosis; M, male; PPRPE, preserved para-arteriolar retinal pigment epithelialium.

* Two children who cannot use the logMAR visual chart were excluded.

# The two children in the non-LCA group were diagnosed with RP.

✝ FFA was preformed on eight children.

**Figure 2.**
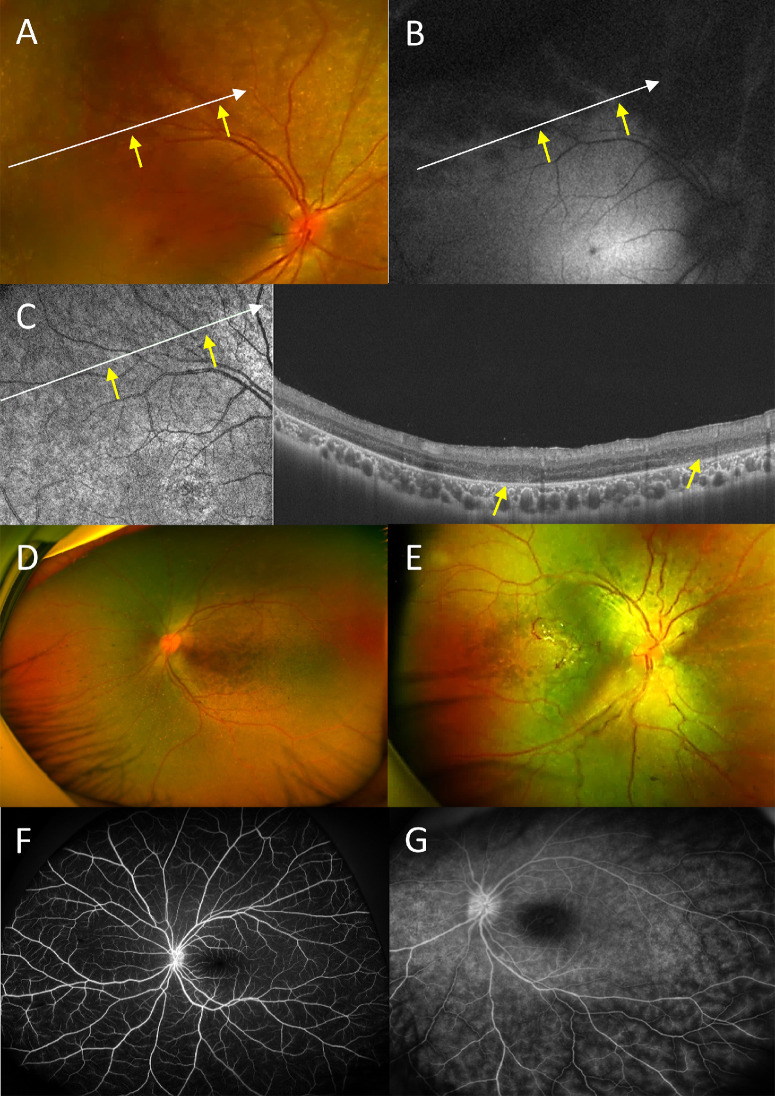
Imaging in CRB1-eoRD. (**A–C**) These panels show the multimodal images of preserved para-arteriolar retinal pigment epithelium (PPRPE), one of the most characteristic features of CRB1 associated RD. Scanning laser ophthalmoscope (**A**) and the ultra-widefield autofluorescence (**B**) showed the retention of autofluorescence along the retinal arteriole (*yellow angle*). SS-OCT clearly revealed the residue of ONL structure beside the artery, which is related to the normal autofluorescence (*yellow angle*), and the fuzzy signal of ONL in the other area, which is related to the dim autofluorescence (*red angle*, and the *white angle* showed the area of OCT B-scan, the *yellow angle* showed the same artery among multimodal imaging). Coin-like yellow-white retinal spots (**D** and **E**) were common fundus characteristics in CRB1-eoRD. The early phases FFA image (**F**) and venous phase FFA image (**G**) in patient ID 10, which revealed the high fluorescein of optic disc and the fern-like vascular leakage at venous phases (**G**).

Coin-like yellow-white retinal spots were present in 20 (90.9%) eyes, except for one LCA child. Atrophic macula was noted in 13 (59.1%) eyes in 7 children with LCA. Preserved para-arteriolar retinal pigment epithelialium (PPRPE) was noted in 12 eyes (54.5%) in 6 children, 5 with LCA and 1 with RP. Cystic macular lesions was in six eyes (27.3%, 1 LCA and 2 RP). Coats-like retinopathy was found in one eye (4.5%, 1 LCA). Fluorescein fundus angiography (FFA) was performed in four children, including two with LCA and two with RP. All eyes (8/8) had fern-like vascular leakage which coud contributed by both RPE staining and vascular leakage (Fig. 2).

The fERG was successfully performed in six children. The scotopic responses and photopic responses were completely extinguished in five children. One 7-year-old boy with bilateral cystic macular lesions showed nearly normal scotopic responses (86.2 ms and 20.3 µV in the right eye and 88.7 ms and 20.3 µV in the left eye in DA 0.01 ERG following 30 minutes in dark adaptation, 0.0 cd/m^2^ background) and decreased photopic responses (a-wave: 14.2 ms and −7.6 µV, b-wave: 34.7 ms and 14.7 µV in right eye and a-wave: 12.5 ms and −6.2 µV, b-wave: 36.5 ms and 12.3 µV in right eye in LA 3 ERG, 0.0 cd/m^2^ background) in both eyes.

### SS-OCT and SS-OCTA Analysis

**Figure 3.**
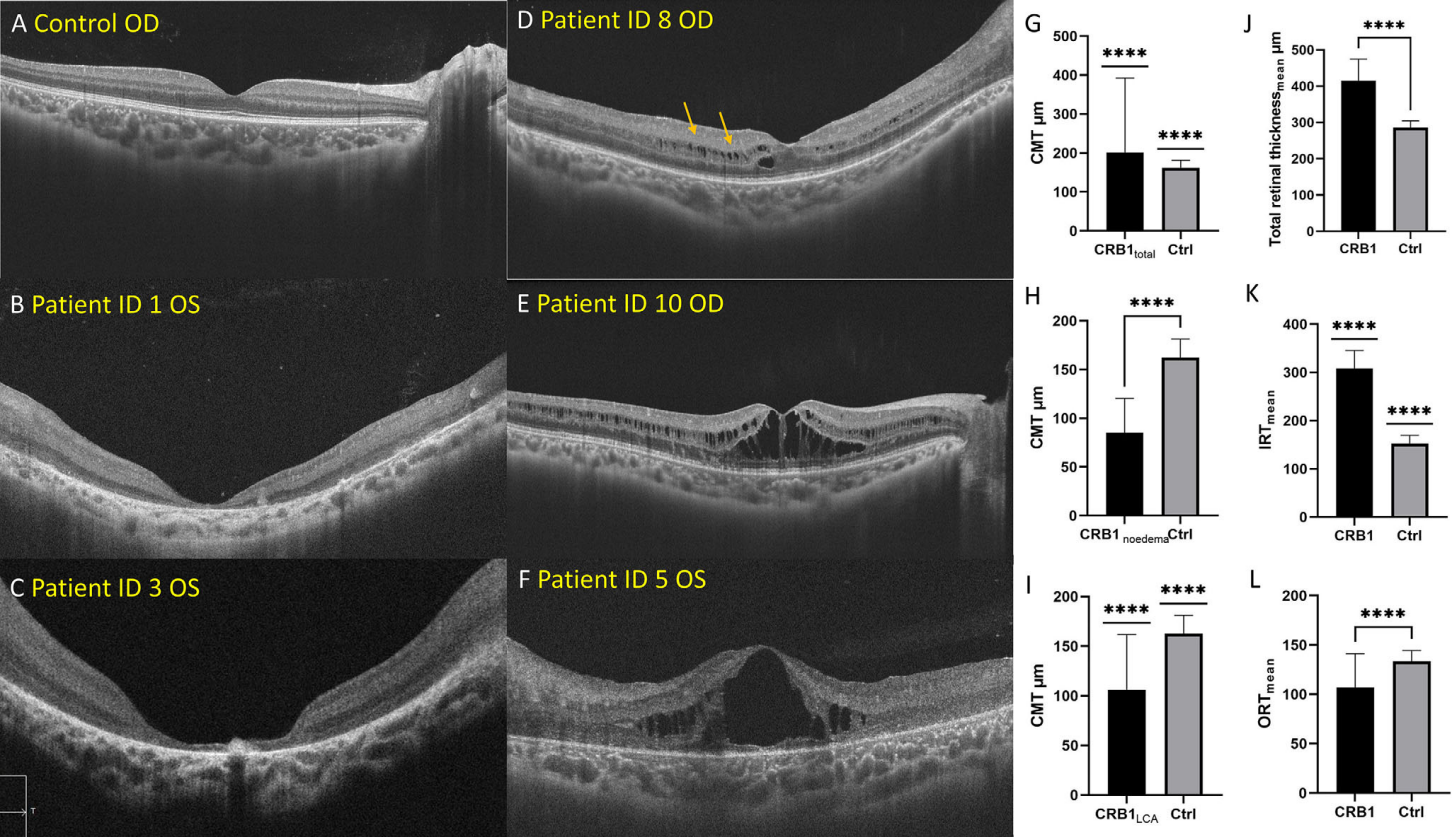
SS-OCT characteristic of CRB1-eoRD. Compared to the healthy control (**A**), atrophic macula (**B, C**) was children with severe LCA, and cystic macular lesion (**D–F**) were present from mild to severe. Central macular thickness in CRB1 group was significantly thicker than control (**G;** *P* < 0.0001), however, the CMT was thinner than control group when excluded macular edema eyes (**H**; *P* < 0.0001) or non-LCA group (**I**; *P* < 0.0001). Total retinal thickness and inner retinal thickness in the CRB1-eoRD were significantly thicker than the control (**J** and **K**; *P* < 0.0001). Outer retinal thickness in the CRB1 were significantly thinner than the control (**L**; *P* < 0.0001).

SS-OCT examination was successfully performed for all children. Outer retinopathy in different stage was shown by SS-OCT in CRB1-eoRD (Fig. 3). Table 2 showed the SS-OCT qualitative evaluation of the outer layer of the retina. Among them, 22.7% of the children's eyes' with ONL were totally atrophic, 31.3% had residual macula, 18.4% had thinning of the ONL, and 27.6% had cystic macular lesion. Only 9.2% retained the integrity of ELM and EZ, 45.4% showed discontinuity of ELM and EZ, and 45.4% ELM and EZ were unrecognizable. The RPE at the macula of CRB1 was mostly intact (72.7%), and 27.3% RPE was interrupted.

To quantitatively analyze the retinal microstructure, the thickness of each retinal layer in CRB1-eoRD was manually measured. We found differences in the thickness of retinal layers between CRB1 and the control group. Overall, the CMT was significantly thicker in CRB1 children compared with the healthy control (194.5 µm vs. 162.6 µm, *P* < 0.0001) and IRDs control (194.5 µm vs. 116.3 µm, *P* < 0.0001). However, subgroup analysis shows the CMT in the LCA group is thinner, instead of thicker, than the healthy control (107.2 µm vs. 162.6 µm, *P* < 0.0001). If excluding the cases with cystic macular lesions (1 with LCA and 2 with RP), CMT is also significantly thinner than the healthy control (89.6 µm vs. 162.6 µm, *P* < 0.0001). Furthermore, the CRB1 group presents a significantly thicker retina with increased total retinal thickness in both nasal and temporal areas (454.4 µm vs. 281.8 µm in healthy control vs. 244.7 µm in IRDs control, 355.9 µm vs. 270.6 µm in healthy control vs. 213.9 µm in IRDs control, 500.0 µm vs. 305.4 µm in healthy control vs. 237.2 µm in IRDs control, 350.4 µm vs. 286.8 µm in healthy control vs. 193.8 µm in IRDs control in N_1.5_, T_1.5_, N_3.0_, and T_3.0_, respectively, *P* < 0.0005). Because the boundary between OPL and ONL is undistinguishable in most cases, the thickness of OPL and ONL was not measured.

To clarify the detail of retinal thickening, ORT and IRT were measured. IRT was significantly thicker than other IRDs and control (both *P* < 0.0001). However, the ORT was significantly thicker than other IRDs (119.8 µm vs. 78.4 µm, *P* = 0.0025) but thinner than the control (119.8 µm vs. 133.4 µm, *P* = 0.0004), and the inner/outer (I/O) ratio was significantly higher in the CRB1-eoRD compared with other IRDs (3.0 vs. 2.2, *P* = 0.0027) and control (3.0 vs. 1.2, *P* < 0.0001). In terms of each layer of the retinal inner layer, the thickness was significantly higher in RNFL, GCL, IPL, and INL than the healthy control, in which RNFL increased the most in N_3.0_ (104.4 µm vs. 20.3 µm, *P* < 0.0001), and GCL, IPL, and INL increased the most in N_1.5_ (85.2 µm vs. 41.8 µm, 73.8 µm vs. 30.3 µm, 108.4 µm vs. 33.9 µm, *P* < 0.0001, respectively). The detailed data are presented in Table 3.

**Figure 4.**
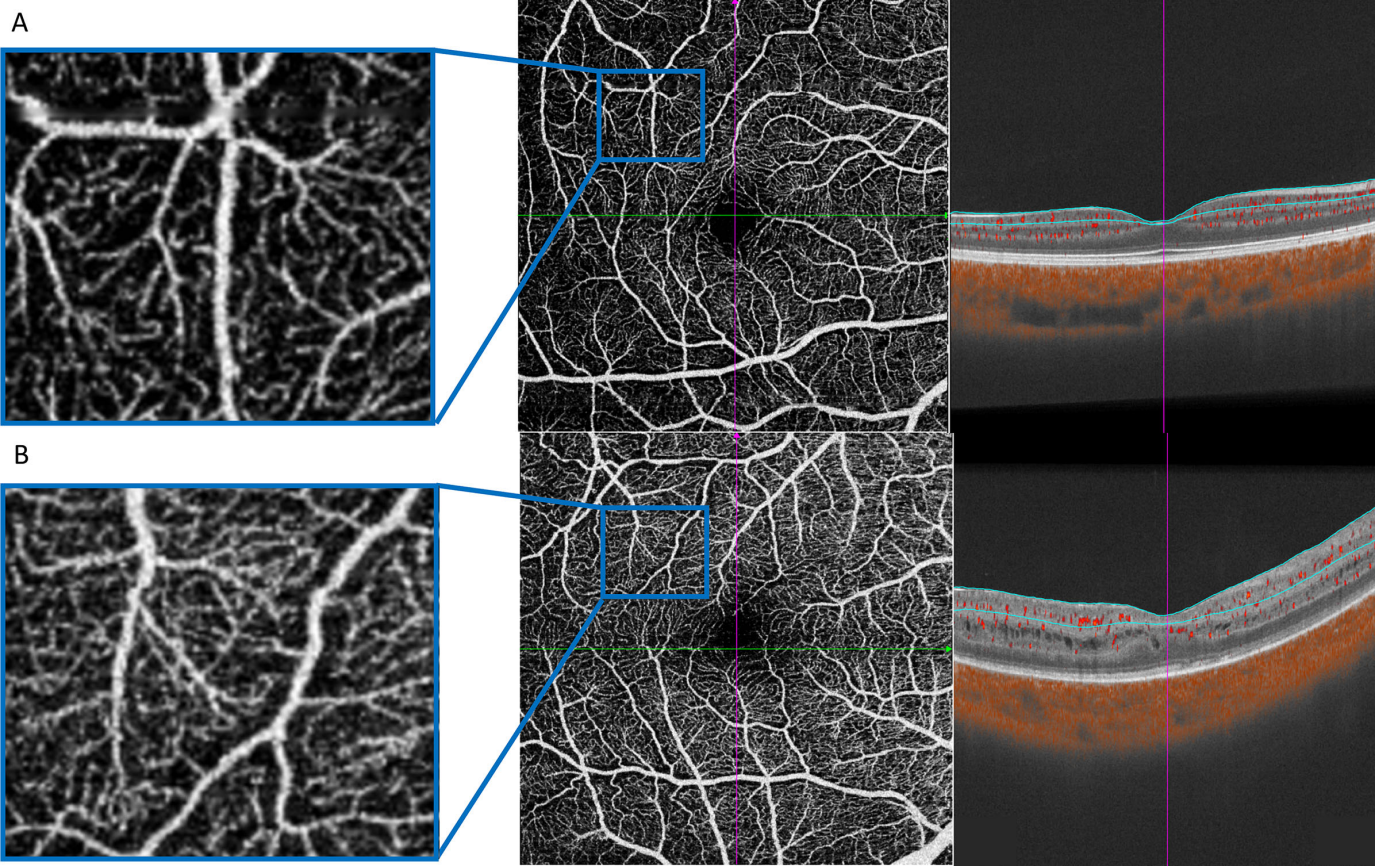
SS-OCTA characteristic about superficial vascular complex in control (**A**) and CRB1-eoRD (**B**). The vascular density and perfusion area of the superficial vascular complex in CRB1 was larger than the control.

SS-OCTA was successfully conducted in two CRB1-eoRD (2 RP). Compared with the control, the vascular density and perfusion area in SVC were higher than control in the central (1 mm), para (1 mm-3 mm), and peri-macular area (3 mm-6 mm; Fig. 4). In DVC, the vascular density and perfusion area were higher in the central macular area but not in the para and peri-macular area (Table 4).

### Genetics

**Figure 5.**
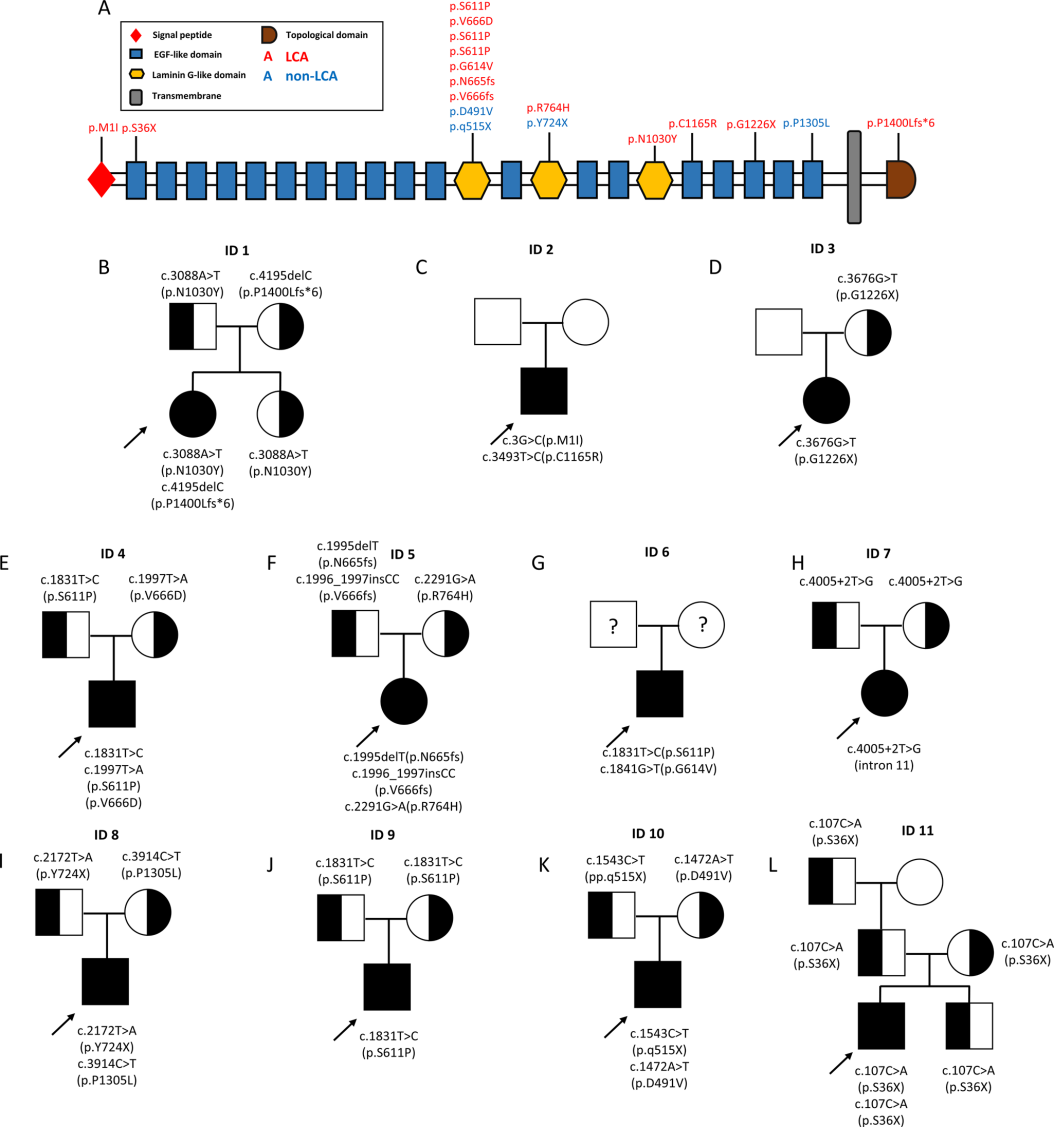
A schematic drawing of the crumbs homologue 1 (CRB1) protein structure and the variants found in this case series. Each variant is detailed above the protein, with its corresponding location, and with different colors according to the associated phenotype (**A**). Pedigrees of families with CRB1-associated early onset RD, Leber congenital amaurosis (LCA; **B–H, J, L**) and non-LCA (**I** and **K**).

Nineteen different *CRB1* variants were detected in our case series, including 12 missense (63%), 3 frameshifts (16%), 3 nonsense (16%), and 1 splicing (5%). Of them, 12 (63%) variants had been reported, and 7 (27%) were novel (Table 5). The c.3088A>T, a novel missense that was predicted as likely pathogenic, was identified in a 5-year-old child with macular atrophy, PPRPE, and yellow-white spots. The SIFT, Poly-Phen 2, MutationTaster, and PROVEAN were predicted as all deleterious. The c.3G>C, a novel missense that was predicted as pathogenic, was identified in a 4-year 6-month old child with LCA. The SIFT, Poly-Phen 2, MutationTaster, and PROVEAN were predicted as deleterious, deleterious, disease causing, and neutral, respectively. The 5 missense mutations c.1831T>C, c.1997T>A, c.1841G>T, c.3914C>T, and c.1472A>T, which were predicted as likely pathogenic or variant of uncertain significance, were reported in the literature.^20^^–^^26^ The three novel frameshift and two nonsense mutations were predicted as pathogenic. Other detailed information on genotype is shown in Table 5. CRB1 protein structure, variants distribution, and pedigree graphs are shown in Figure 5. There was no significant correlation between pathogenic variants and phenotype.

**Table 2.** SS-OCT Retinal Outer Layer Qualitative Analysis of CRB1-Associated Early Onset Retinal Dystrophy

OCT Retinal Outer Layer Characteristics (Eyes)					
ONL	Totally atrophic	Residual macula		Thinning	Fovea cystoid changes
	5/22 (22.7%)	7/22 (31.3%)		4/22 (18.4%)	6/22 (27.6%)
ELM	Intact		Interrupted		Unrecognizable
	2/22 (9.2%)		10/22 (45.4%)		10/22 (45.4%)
EZ	Intact		Interrupted		Unrecognizable
	2/22 (9.2%)		10/22 (45.4%)		10/22 (45.4%)
RPE at the macula	Intact		Interrupted		Unrecognizable
	16/22 (72.7%)		6/22 (27.3%)		0/22 (0%)

ELM, external limiting membrane; EZ, ellipsoid zone; ONL, outer nuclear layer; RPE, retinal pigment epithelium; SS-OCT, swept-source optical coherence tomography.

**Table 3.** SS-OCT Retinal Quantitative Analysis of CRB1-Associated Early Onset Retinal Dystrophy

		CRB1	Other IRDs	Control	*P* Value_crb1_ Vs. other IRDs	*P* Value_crb1_ Vs. Control	ANOVA *P* Value
CMT	Total	194.5 ± 184.5	116.3 ± 63.03	162.6 ± 18.7	<0.0001	<0.0001	<0.0001
	Excluded edema	89.6 ± 33.5	116.3 ± 63.03	162.6 ± 18.7	<0.0001	<0.0001	<0.0001
	LCA group	107.2 ± 53.5	116.3 ± 63.03	162.6 ± 18.7	<0.0001	<0.0001	<0.0001
Total Retinal thickness	N_1.5_	454.2 ± 98.6	244.7 ± 84.02	281.8 ± 24.0	<0.0001	<0.0001	<0.0001
	T_1.5_	355.9 ± 45.1	213.9 ± 73.34	270.6 ± 22.5	<0.0001	<0.0001	<0.0001
	N_3.0_	500.0 ± 109.0	237.2 ± 84.38	305.4 ± 19.8	<0.0001	<0.0001	<0.0001
	T_3.0_	350.4 ± 39.5	193.8 ± 66.88	286.8 ± 18.8	<0.0001	0.0005	<0.0001
	Total mean	415.1 ± 57.4	242.6 ± 72.65	286.2 ± 18.2	<0.0001	<0.0001	<0.0001
ORT	N_1.5_	133.5 ± 64.4	73.8 ± 33.43	134.8 ± 19.0	<0.0001	0.9081	<0.0001
	T_1.5_	103.0 ± 27.7	78.1 ± 31.28	136.7 ± 10.8	0.0199	<0.0001	<0.0001
	N_3.0_	134.0 ± 75.5	77.1 ± 35.03	136.6 ± 17.7	0.0006	0.0570	<0.0001
	T_3.0_	100.1 ± 28.7	78.7 ± 33.47	129.9 ± 10.7	0.0176	0.0002	<0.0001
	Total mean	119.8 ± 40.5	78.4 ± 28.34	133.4 ± 10.9	0.0025	0.0004	<0.0001
IRT	N_1.5_	332.8 ± 66.3	187.2 ± 24.83	147.1 ± 20.8	<0.0001	<0.0001	<0.0001
	T_1.5_	261.6 ± 27.7	155.2 ± 21.57	135.1 ± 16.4	<0.0001	<0.0001	<0.0001
	N_3.0_	374.9 ± 70.7	181.7 ± 40.87	171.0 ± 22.6	<0.0001	<0.0001	<0.0001
	T_3.0_	264.1 ± 28.6	132.8 ± 27.35	158.0 ± 15.4	<0.0001	<0.0001	<0.0001
	Total mean	299.3 ± 37.3	164.2 ± 23.48	152.8 ± 16.3	<0.0001	<0.0001	<0.0001
I/O ratio	N_1.5_	3.0 ± 1.4	2.7 ± 1.06	1.1 ± 0.3	0.7046	<0.0001	<0.0001
	T_1.5_	3.1 ± 0.9	2.3 ± 0.82	1.0 ± 0.1	0.0004	<0.0001	<0.0001
	N_3.0_	3.5 ± 1.0	2.8 ± 1.27	1.3 ± 0.3	0.0362	<0.0001	<0.0001
	T_3.0_	2.9 ± 1.1	2.0 ± 0.82	1.2 ± 0.2	0.0002	<0.0001	<0.0001
	Total mean	3.0 ± 1.1	2.2 ± 0.65	1.2 ± 0.2	0.0027	<0.0001	<0.0001
RNFL	N_1.5_	46.0 ± 20.2	32.0 ± 11.16	13.6 ± 2.9	0.0110	<0.0001	<0.0001
	T_1.5_	23.0 ± 11.7	16.3 ± 6.85	10.4 ± 1.0	0.3812	0.0024	0.0035
	N_3.0_	104.4 ± 33.0	60.2 ± 14.53	20.3 ± 7.1	0.0001	<0.0001	<0.0001
	T_3.0_	26.0 ± 15.9	10.1 ± 12.72	11.3 ± 2.9	<0.0001	0.0228	<0.0001
	Total mean	49.8 ± 15.5	29.6 ± 8.14	13.9 ± 2.5	<0.0001	<0.0001	<0.0001
GCL	N_1.5_	85.2 ± 21.8	61.6 ± 15.49	41.8 ± 11.9	0.0004	<0.0001	<0.0001
	T_1.5_	65.2 ± 22.1	47.6 ± 11.66	44.1 ± 15.1	0.0290	0.0070	0.0046
	N_3.0_	70.3 ± 22.9	38.4 ± 14.62	61.1 ± 11.3	<0.0001	0.4319	<0.0001
	T_3.0_	64.1 ± 15.6	41.7 ± 14.04	51.7 ± 9.3	<0.0001	0.0960	<0.0001
	Total mean	71.2 ± 13.8	47.3 ± 9.36	49.7 ± 7.7	<0.0001	<0.0001	<0.0001
IPL	N_1.5_	73.8 ± 22.3	45.3 ± 10.02	30.3 ± 6.1	<0.0001	<0.0001	<0.0001
	T_1.5_	65.9 ± 21.7	47.3 ± 11.88	33.3 ± 7.7	0.0031	<0.0001	<0.0001
	N_3.0_	66.5 ± 21.1	40.1 ± 8.62	42.6 ± 2.9	<0.0001	0.0004	<0.0001
	T_3.0_	74.2 ± 20.4	44.6 ± 11.24	43.9 ± 4.5	<0.0001	<0.0001	<0.0001
	Total mean	70.1 ± 16.6	44.6 ± 11.24	37.5 ± 3.0	<0.0001	<0.0001	<0.0001
INL	N_1.5_	108.4 ± 58.8	48.3 ± 6.79	33.9 ± 4.3	<0.0001	<0.0001	<0.0001
	T_1.5_	83.7 ± 29.6	44.0 ± 7.86	37.1 ± 5.0	<0.0001	<0.0001	<0.0001
	N_3.0_	98.5 ± 17.7	43.0 ± 13.30	47.7 ± 9.2	<0.0001	<0.0001	<0.0001
	T_3.0_	84.4 ± 21.4	36.4 ± 10.86	43.7 ± 5.2	<0.0001	<0.0001	<0.0001
	Total mean	93.8 ± 20.3	42.9 ± 6.72	40.6 ± 4.5	<0.0001	<0.0001	<0.0001

CMT, central macular thickness; GCL, ganglion cell layer; INL, inner nucleus layer; I/O ratio, inner layer/ outer layer ratio; IPL, inner plexiform layer; IRDs, inherited retinal diseases; IRT, inner retinal thickness; LCA, Leber congenital amaurosis; N_1.5_, 1.5mm nasal to macular; N_3.0_, 3.0 mm nasal to macular; ORT, Outer retinal thickness; RNFL, retinal nerve fiber layer; SS-OCT, swept-source optical coherence tomography; T_1.5_, temporal 1.5 mm to macular; T_3.0_, 3.0 mm temporal to macular.

Other IRDs group, including 11 age-matched inherited retinal diseases with various genetic variants, such as ABCA4, RDH12, RPGR, LCA5, CEP290, RP2, USH2A, and EYS but without CRB1 variant as the control group.

**Table 4.** SS-OCTA Retinal Quantitative Analysis of Early Onset CRB1-Associated Early Onset Retinal Dystrophy

			CRB1^*^	Control
Superficial vascular complex	Vascular density (%)	0-1 mm	28.0 ± 6.7	14.2 ± 2.8
		1-3 mm	54.4 ± 4.4	44.0 ± 5.3
		3-6 mm	59.0 ± 4.2	41.8 ± 4.4
	Perfusion area (mm^2^)	0-1 mm	0.3 ± 0.03	0.1 ± 0.02
		1-3 mm	3.4 ± 0.14	2.7 ± 0.27
		3-6 mm	12.3 ± 0.53	8.8 ± 0.70
Deep vascular complex	Vascular density (%)	0-1 mm	41.1 ± 6.3	27.8 ± 3.4
		1-3 mm	32.6 ± 7.3	53.4 ± 2.0
		3-6 mm	25.5 ± 1.9	54.1 ± 1.9
	Perfusion area (mm^2^)	0-1 mm	0.3 ± 0.02	0.2 ± 0.03
		1-3 mm	2.0 ± 0.10	3.1 ± 0.12
		3-6 mm	6.4 ± 0.43	10.4 ± 0.30

SS-OCT, swept-source optical coherence tomography angiography.

* Low visual acuity in CRB1 phenotyped as LCA hinder SS-OCTA examination.

**Table 5.** Causative Variants in the 11 Probands

													ACMG Classification						
														Identified Classification Rules					
ID	Diagnoses	Nucleotide Change	Protein Changes	Position	Type	Co-Segregation	Exon	SIFT	Poly-Phen 2	MutationTaster	PROVEAN	CADD	Verdict	Criterion 1	Criterion 2	Criterion 3	Criterion 4	Criterion 5	Source
1	LCA	c.3088A>T	p.N1030Y	chr1:197404081	Missense	Paternal	9	Deleterious	Deleterious	DC	Deleterious	0.53	Likely pathogenic	PM2	PM3	PP3	PP4		Novel
		c.4195delC	p.P1400Lfs*6	chr1:197446982	Frameshift	Maternal	12	NA	NA	NA	NA	NA	Pathogenic	PVS1	PM2	PP4			Novel
2	LCA	c.3G>C	p.M1I	chr1:197237545	Missense	/	1	Deleterious	Deleterious	DC	Neutral	0.35	VUS	PM2	PM3	PP4			Novel
		c.3493T>C	p.C1165R	chr1:197404486	Missense	/	7	Deleterious	Deleterious	DC	Deleterious	0.69	Likely pathogenic	PM2	PM3	PM5	PP3	PP4	Known
3	LCA	c.3676G>T	p.G1226X	chr1:197404669	Missense	Maternal	9	NA	NA	DC	NA	0.99	Pathogenic	PVS1	PM3	PM2			Known
4	LCA	c.1831T>C	p.S611P	chr1:197390789	Missense	Maternal	6	Tolerated	Deleterious	DC	Deleterious	0.74	Pathogenic	PM3	PM2	PP3	PP4		Known
		c.1997T>A	p.V666D	chr1:197390955	Missense	Paternal	6	Deleterious	Deleterious	DC	Deleterious	0.64	Likely pathogenic	PM3	PM2	PP3	PP4		Known
5	LCA	c.1995delT	p.N665fs	chr1:197390952	Frameshift	Maternal	6	NA	NA	NA	NA	NA	Pathogenic	PVS1	PM2	PM3			Novel
		c.1996_1997insCC	p.V666fs	chr1:197390954	Frameshift	Paternal	6	NA	NA	NA	NA	NA	Pathogenic	PVS1					Novel
		c.2291G>A	p.R764H	chr1:197396746	Missense	Maternal	7	Tolerated	Deleterious	NA	NA	0.34	Pathogenic	PM3	PM2	PM5	PP3	PP4	Known
6	LCA	c.1831T>C	p.S611P	chr1:197390789	Missense	NA	6	Tolerated	Deleterious	DC	Deleterious	0.74	Pathogenic	PM3	PM2	PP3	PP4		Known
		c.1841G>T	p.G614V	chr1:197390799	Missense	NA	6	Deleterious	Deleterious	DC	Deleterious	0.92	Pathogenic	PM3	PM2	PP3	PP4		Known
7	LCA	c.4005+2T>G	/	chr1:197411424	Splicing	Paternal/Maternal	Intron11	NA	NA	DC	NA	0.95	Pathogenic	PVS1	PM2	PM3	PP4		Known
8	non-LCA(RP)	c.2172T>A	p.Y724X	chr1:197396627	Nonsense	Paternal	7	NA	NA	NA	NA	NA	Pathogenic	PVS1	PM3	PM2	PP4		Known
		c.3914C>T	p.P1305L	chr1:197411331	Missense	Maternal	11	Deleterious	Benign	DC	Deleterious	0.63	Pathogenic	PM3	PM5	PM2	PP3		Known
9	LCA	c.1831T>C	p.S611P	chr1:197390789	Missense	Paternal/Maternal	6	Tolerated	Deleterious	DC	Deleterious	0.41	Pathogenic	PM3	PM2	PP3	PP4		Known
10	non-LCA(RP)	c.1472A>T	p.D491V	chr1:197390430	Missense	Maternal	6	Tolerated	Benign	DC	Deleterious	0.22	VUS	PM2	PM3				Known
		c.1543C>T	p.q515X	chr1:197390501	Nonsense	Paternal	6	NA	NA	NA	NA	0.96	Pathogenic	PVS1	PM2	PP4			Novel
11	LCA	c.107C>A	p.S36X	chr1:197297588	Nonsense	Paternal/ Maternal	2	NA	NA	NA	NA	NA	Pathogenic	PVS1	PM2	PM3	PP4		Novel

DC, disease causing; LCA, Leber congenital amaurosis; NA, none available; VUS, variant of uncertain significance.

## Discussion

CRB1-associated retinal dystrophies (CRB1-RDs) encompass a wide range of phenotypes, including LCA/EOSRD, RP, and MD, which may present as poor vision, nystagmus, hyperopia, and night blindness. CRB1-RD exhibits unique and diverse features, such as PPRPE, coin-like yellow-white spots, retinal thickening, retinal telangiectasia (coats-like), and intermediate uveitis.^27^^–^^29^ In this study, we provided a comprehensive analysis of the genotypic and phenotypic characteristics, particularly focusing on the qualitative and quantitative aspects of CRB1-eoRD in a cohort of very young children, with a mean age of 4.82 years old (1.67–7.0 years old). Given the higher proportion of LCA and the rarity of MD phenotypes among children, we categorized them into two groups: LCA and non-LCA (including RP and MD). Notably, 81.8% of the children were diagnosed with LCA, with a mean BCVA of 1.50 ± 0.87. Compared to previous studies in CRB1 related IRD in the general population,^3^^,^^13^^,^^30^ our study observed a higher proportion of LCA and relatively lower BCVA. This finding underscores the importance of considering an early treatment for forthcoming *CRB1* gene therapy trials.

Retinal thickness changes have been a key feature reported in CRB1-RD. To measure retinal thickness preciously, we manually measured by a single investigator, and to minimize potential error, each measurement was taken three times. In our study, we opted to match controls based on age and sex to minimize potential confounding factors. We realized that matching for refractive error or axial length could further enhance the precision of our comparisons. However, it is difficult to match the refractive error around +6 D in healthy controls. Whereas previous studies measured retinal thickness using an automatic segmentation and measurement system,^3^ our high-resolution SS-OCT revealed thickening in the IRT in CRB1-eoRD. Our study reported a higher proportion of retinal inner layer thickening (100%) compared to previous reports (78% to 95%).^2^^–^^4^^,^^13^ Interestingly, the ORT in CRB1-eoRD was thicker than other IRDs group but thinner than the control group. Additionally, we introduce the novel OCT parameter, I/O ratio, which was increased in 100% (22/22) of patients with CRB1-eoRD, which significantly exceeded that of the other IRDs group (3.0 vs. 2.2, *P* = 0.0027) and the control group (3.0 vs. 1.2, *P* < 0.0001). The other IRD group included Stargardt's disease carrying ABCA4 variants, as well as individuals with Cone-Rod Dystrophy (CORD) or RP presenting with various genetic variants such as RDH12, RPGR, LCA5,CEP290, RP2, USH2A, and EYS. The I/O ratio highlights relative inner retina thickening and outer retinal thinning, and may be helpful for the diagnosis of different IRDs. The implications of this marker in genotype-phenotype correlation require further exploration in larger patient cohorts, encompassing patients with a broader age range and other genetic diagnoses. Notably, every layer of the inner retina exhibited thickening, a unique retinal architecture resulting from incomplete apoptosis of the retinal inner layers and dysplasia of the retinal outer layers.^10^ Cho SH et al.^31^ reported in their study involving mouse/human models that the CRB1 protein plays a pivotal role in the maintenance of adherens junctions within retinal cells, with expression observed on Müller glial cells. Notably, Crb1/Crb2 knockout mice exhibited an increase in the population of Müller glial cells. Therefore, it is plausible that the observed thickening of the inner retinal layer may be attributed to the proliferation of Müller cells and/or incomplete apoptosis within the retinal inner layers.

Another major characteristic of CRB1-RD is the loss of retinal lamination. Although we noted ill-defined lamination of the outer layer, all patients exhibited identifiable inner layers, distinguishing this feature as characteristic of CRB1-eoRD. This finding contrasts with reports by Talib et al. and Varela et al., which described ill-defined or disorganized retinal lamination in over 50% of patients.^3^^,^^13^ Our data provided the evidence that 77.3% of children still have residual outer retinal structure, which may be amenable to genetic rescue. The insights gained from this study hold significant value in determining the optimal therapeutic window for potential *CRB1* gene therapy trials in the near future. Meanwhile, thickening was observed in every layer of the inner retina in CRB1-eoRD, including RNFL, GCL, IPL, and INL compared to controls, contributing to our understanding of the disease pathogenesis. Larger patient cohorts are warranted to further verify these characteristics, especially quantifying residual photoreceptors in CRB1-eoRD.

Intriguingly, the two non-LCA children in our study presented with cystic macular lesions and vasculitis, initially misdiagnosed as intermediate uveitis. This observation underscores that IRD should be considered in young children with unexplained cystic macular lesions. Furthermore, we successfully performed SS-OCTA in these non-LCA children, analyzing the retinal macrovascular structure for the first time in these cases. Surprisingly, we found higher vascular density and perfusion area in both the SVC and DVC at 1 to 3 mm and 3 to 6 mm, which contrasts with a recent study reporting decreased vascular density and perfusion area in both SVC and DVC.^32^ However, the phenotypic distribution in that study was not described, limiting direct comparison with our findings. Future research with enriched SS-OCTA data of CRB1-RD is warranted to analyze OCTA characteristics in different phenotypes (LCA, RP, and MD).

In the present study, we also identified 7 novel mutations of CRB1 in 11 unrelated CRB1-eoRD families. Three of seven were frameshift mutations, two were missense mutations, and two were nonsense mutation. Patient IDs 8 and 10, with the heterozygous missense mutation and nonsense mutation, presented as a mild RP. In patients with LCA, the most common mutations were missense and frameshift mutations. Given the clinical heterogeneity of CRB1-RD, further exploration is needed to establish genotype-phenotype correlations.

Whereas our study contributes valuable insights into CRB1-RD in childhood and introduces novel retinal microstructure findings using high-resolution SS-OCT, it also has several limitations. First, its retrospective nature and relatively small case series, particularly in the non-LCA group, may introduce selection bias. Second, due to limitations inherent in existing detection technology, we were able to obtain reliable OCTA data from only two patients with non-LCA phenotypes. Several factors contributed to the low detection rate, including issues such as nystagmus (3/11, 27.3%), young age (4/11, 36.4%), and fixation instability in patients with low visual acuity (2/11, 18.2%). Consequently, we acknowledge the need for further research to investigate retinal microvascular changes in a larger patient cohort. Last, the cross-sectional study design limits our ability to assess long-term disease progression. However, we plan to address these limitations in future research by expanding the CRB1-eoRD cohort and conducting longitudinal follow-ups to observe the natural history of the disease.

In conclusion, our study provides a comprehensive report on the genotypic and phenotypic characteristics of CRB1-eoRD, particularly highlighting the qualitative and quantitative analysis of SS-OCT. The novel biomarker, I/O ratio, may facilitate early diagnosis for CRB1-eoRD. The detailed description of SS-OCTA features contributes to a comprehensive understanding of the disease. Furthermore, the insights gained from this study hold significant value in determining the optimal therapeutic window for potential *CRB1* gene therapy trials in the near future. Our study deepens the understanding of CRB1-eoRD and expands the knowledge of this disease.
